# A Human Deciduous Tooth and New 40Ar/39Ar Dating Results from the Middle Pleistocene Archaeological Site of Isernia La Pineta, Southern Italy

**DOI:** 10.1371/journal.pone.0140091

**Published:** 2015-10-12

**Authors:** Carlo Peretto, Julie Arnaud, Jacopo Moggi-Cecchi, Giorgio Manzi, Sébastien Nomade, Alison Pereira, Christophe Falguères, Jean-Jacques Bahain, Dominique Grimaud-Hervé, Claudio Berto, Benedetto Sala, Giuseppe Lembo, Brunella Muttillo, Rosalia Gallotti, Ursula Thun Hohenstein, Carmela Vaccaro, Mauro Coltorti, Marta Arzarello

**Affiliations:** 1 Dipartimento Studi Umanistici, Sezione di Scienze Preistoriche e Antropologiche, Università degli Studi di Ferrara, LT, TekneHub, Ferrara, Italy; 2 Dipartimento di Biologia, Laboratorio di Antropologia, Università di Firenze, Firenze, Italy; 3 Dipartimento di Biologia Ambientale, Sapienza Università di Roma, Rome, Italy; 4 Laboratoire des Sciences du Climat et de L’Environnement UMR 8212, IPSL-CEA-CNRS-UVSQ, Gif sur Yvette, France; 5 Ecole française de Rome, Rome, Italy; 6 UMR 7194 – Département de Préhistoire du Muséum national d’Histoire naturelle, Paris, France; 7 Dipartimento di Scienze dell'Antichità, Sapienza Università di Roma, Rome, Italy; 8 Université Bordeaux 1, UMR 5199 PACEA-PPP, Talence, France; 9 Dipartimento di Fisica e Scienze della Terra, Università degli Studi di Ferrara, Ferrara, Italy; 10 Dipartimento di Scienze Fisica, della Terra e dell’Ambiente, Siena, Italy; University of California Los Angeles, UNITED STATES

## Abstract

Isernia La Pineta (south-central Italy, Molise) is one of the most important archaeological localities of the Middle Pleistocene in Western Europe. It is an extensive open-air site with abundant lithic industry and faunal remains distributed across four stratified archaeosurfaces that have been found in two sectors of the excavation (3c, 3a, 3s10 in sect. I; 3a in sect. II). The prehistoric attendance was close to a wet environment, with a series of small waterfalls and lakes associated to calcareous tufa deposits. An isolated human deciduous incisor (labelled IS42) was discovered in 2014 within the archaeological level 3 coll (overlying layer 3a) that, according to new ^40^Ar/^39^Ar measurements, is dated to about 583–561 ka, i.e. to the end of marine isotope stage (MIS) 15. Thus, the tooth is currently the oldest human fossil specimen in Italy; it is an important addition to the scanty European fossil record of the Middle Pleistocene, being associated with a lithic assemblage of local raw materials (flint and limestone) characterized by the absence of handaxes and reduction strategies primarily aimed at the production of small/medium-sized flakes. The faunal assemblage is dominated by ungulates often bearing cut marks. Combining chronology with the archaeological evidence, Isernia La Pineta exhibits a delay in the appearance of handaxes with respect to other European Palaeolithic sites of the Middle Pleistocene. Interestingly, this observation matches the persistence of archaic morphological features shown by the human calvarium from the Middle Pleistocene site of Ceprano, not far from Isernia (south-central Italy, Latium). In this perspective, our analysis is aimed to evaluate morphological features occurring in IS42.

## Introduction

The Isernia La Pineta site (south-central Italy, Molise) is an extensive open-air archaeological site of the Lower Palaeolithic. The abundant lithic assemblage and faunal remains are distributed across four archaeosurfaces and two sectors (3c, 3a, 3s10 in sect. I, 3a in sect. II). Abundant lithic industries and faunal remains were recovered associated with the human tooth. The faunal assemblage, characteristic of the Middle Galerian, is represented by *Bison schoetensacki*, *Palaeoloxodon antiquus*, *Stephanorhinus hundsheimensis*, *Hippopotamus* cf. *antiquus*, *Premegaceros solilhacus*, *Cervus elaphus* cf. *acoronatus*, *Dama* cf. *roberti*, *Capreolus* sp., *Sus scrofa*, *Hemitragus* cf. *bonali*. Carnivores are documented only by *Ursus deningeri*, *Panthera pardus* and *Panthera leo fossilis*[1–4]. The intensive and systematic exploitation of herbivore carcasses as a food supply is shown by numerous cut marks and intentional fresh bones fractures [5,6]. The faunal assemblage has been attributed to the Early Toringian by the presence of the small mammals *Arvicola mosbachensis*, *Sorex* aff. *runtonensis*, *Pliomys episcopalis* and *Microtus* (*Terricola*) *arvalidens* [2–4]. The climate during the human occupation was probably more arid and cooler than at present and the environment was characterized by an arboreal steppe [7].

The lithic production found in layer 3 coll is characterized by the exploitation of two local raw materials: flint and limestone. The flint tablets were reduced by direct and bipolar percussion. The *chaines opératoires* are mainly short, as a consequence of the small size of the raw material, and the cores indicate unipolar, multidirectional and centripetal (cf. discoid) exploitation. Bipolar percussion on an anvil was used for the reduction of flint tablets of low quality, characterized by many fractures. The flakes do not have standardized shapes but are generally thick, short and often overflowing. Modification of the cutting edges was adopted mainly for the manufacture of sidescrapers and denticulates.

The local limestone cobbles were exploited mainly for debitage and rarely for shaping. The debitage reduction sequences were aimed at obtaining flakes (with larger dimensions than the ones on flint) by the exploitation of one to three striking platforms by means of a unipolar method. Shaping is evident for flat cobbles in which a functional edge was created by the removal of some flakes.

Isernia La Pineta seems to be slightly younger than the earliest diffusion of the Acheulean technology in Western Europe, which has been so far recorded at La Noira (Central France) and Notarchirico (Southern Italy), both dated around 700 ka [8,9]. Between 700 to 500 ka, we see the co-existence of sites with [8,10,11] and without [12–14] handaxes in Western Europe. However, all those sites have a common debitage substratum and a similar strategy of raw material supply. This dichotomy could be related to several natural/cultural factors (environment, site function, raw material availability, etc.) or to different hominin species [15–19].

In this paper, we describe the first human specimen discovered at this site: a deciduous incisor, which was found in 2014 within the layer 3 coll (Unit 3). It represents at present the oldest hominin of the Italian fossil record [20].

## Geological Settings

**Fig 1:**
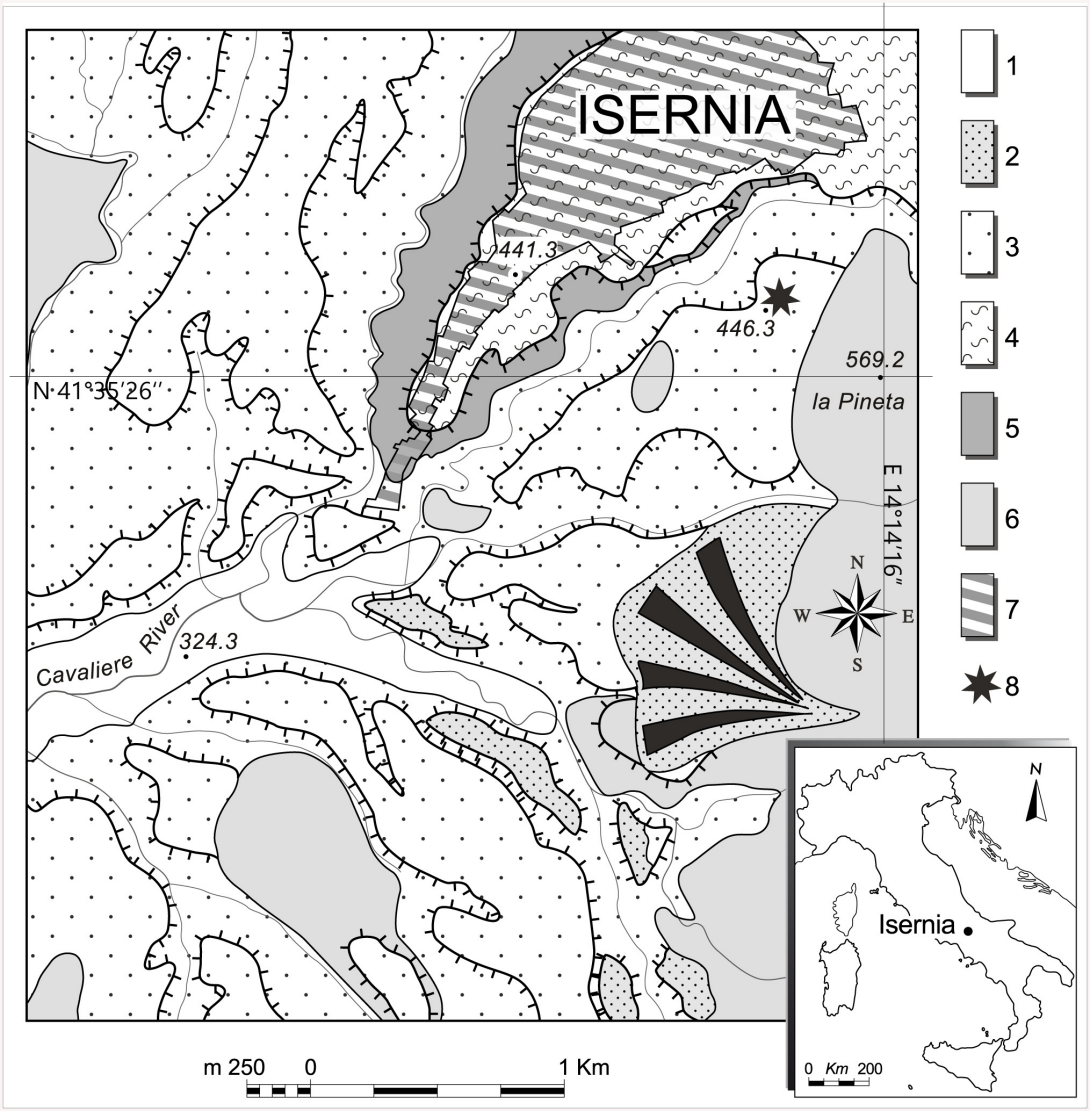
Geographical position of the Isernia La Pineta site. 1. Sandy, gravelly and silty alluvial deposits (Holocene); 2, Sandy, gravelly and silty alluvial deposits (Late Pleistocene); 3, Sandy, gravelly and silt with intercalated pyroclastic layers (Middle Pleistocene); 4, calcareous tufa (Early Middle Pleistocene); 5, silts and clays (Early Pleistocene); 6, bedrock; 7: urbanized area; 8, Isernia la Pineta archaeological site.

Isernia La Pineta is located in south-central Italy (Isernia, Molise) at 457 m above sea level, along the left side of the Cavaliere Stream, in the middle upper part of the Volturno River basin. It has been discovered in the fluvio-lacustrine sedimentary series of “Le Piane basins” during road construction works in 1978 (Fig 1) [21–23]. “Le Piane basins” is a sub-basin within the larger system of extensional intra-montane tectonic basins of Isernia called Carpino-Le Piane Basins Fault System (CLPBFS, [24]). The tectonic activity that generated the Isernia depression is still active and controls the intense seismicity of the area [25,26]. Various eruptions occurred concomitantly with the deposition of the fluvio-lacustrine successions. The volcanic components of fluvio-lacustrine sedimentary series in Southern Apennines are mainly associated with the plinian and ultraplinian eruptions of Roccamonfina and Vulture volcanoes.

The filling of the basin is characterized by four major unconformity bounded stratigraphic units (UBSUs) that deposited during the Pleistocene and Holocene [22,23,27,28] and which are arranged in three terraced fluvial deposits located at progressive elevation on the valley floor (Fig 1). The oldest UBSU (Early to Middle Pleistocene) is made of ca. 60 m thick gravels, silt and clays covered by calcareous tufa up to 20 m in thickness. The successive UBSU (Middle Pleistocene) includes sands, gravels and silts containing a number of coarse grained tephra (pumice) layers that out-crop extensively to the south west of Isernia [29].

In the Isernia town area the calcareous tufa crops out without the following sedimentary cover (Fig 1) but moving eastward, in the Isernia la Pineta area, they are covered by sands and gravels. The top depositional surface of the tufa and that of the alluvial deposits (UBSU 1 and 2) are located almost at the same elevation and were named “main filling” [22,23], or “main unit” [27,28]. The youngest UBSU corresponds to a terrace framed between the Holocene floodplain and the older UBSU and is attributed to the Late Pleistocene [27–29] (Fig 1). Alluvial fans coming from the lateral valleys locally covered the oldest UBSU and laterally correlate with the Late Pleistocene terrace.

**Fig 2:**
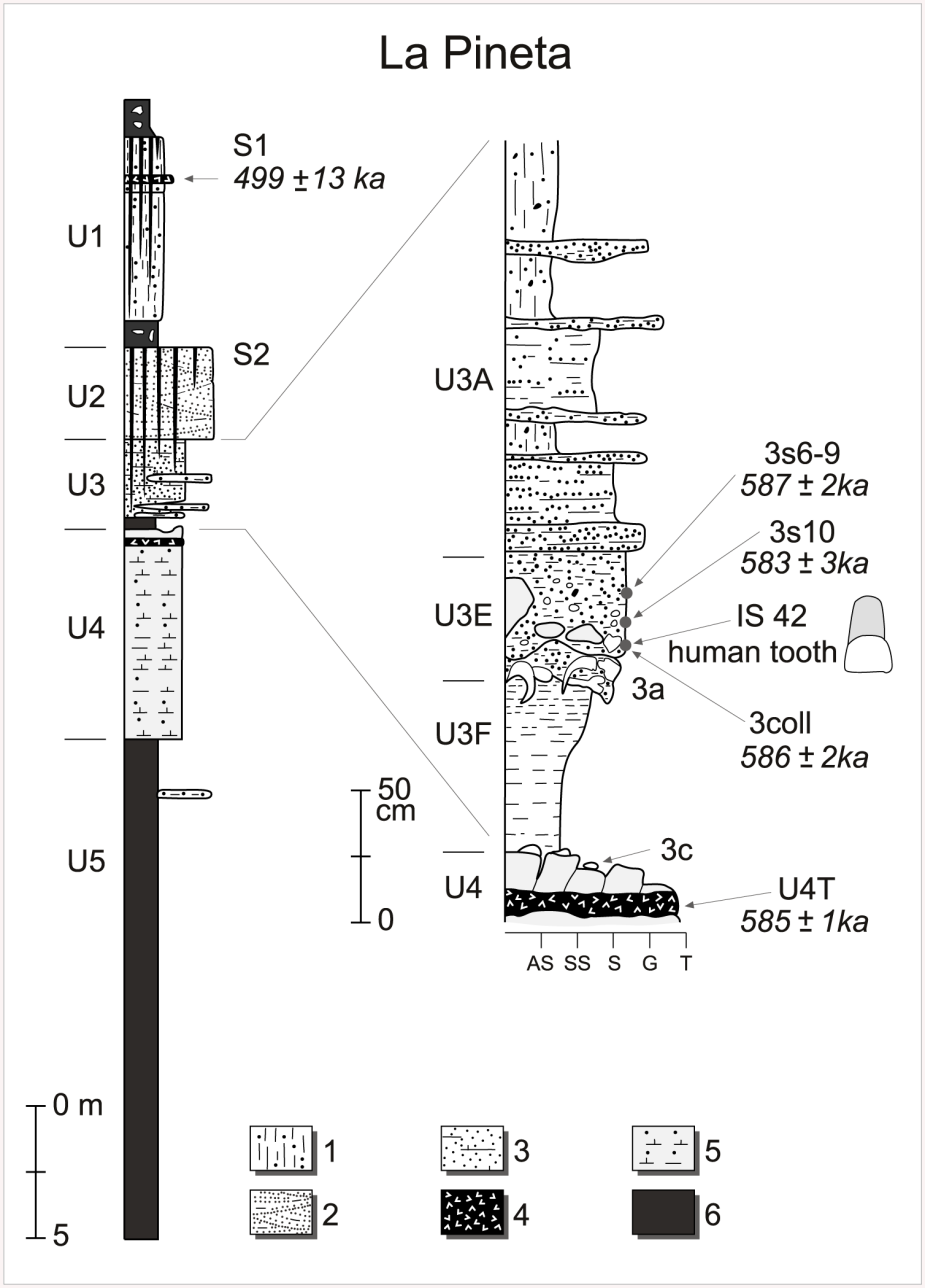
Stratigraphy of Isernia la Pineta site. On the left is shown the general stratigraphy of Isernia la Pineta site characterized by 5 main Units (U1 to 5) and 2 buried palaeosols (S1 and 2). On the right, details on the stratigraphy of Unit 3 that contains various sedimentological layers (U3A, U3E, U3F) including two major archaeological layers (3a and 3c). Bold italic ages (40Ar/39Ar single crystal) are the ones obtained in this study. 1: colluvial deposits; 2: sands and gravels; 3:sands rich in reworked volcanic material and thin layers of gravels; 4: pyroclastic deposits; 5, calcareous tufa; 6: silts and clays. In the left column; AS, silts and clays; SS, silts and fine sands; S, medium to coarse sands; G, gravels; T, calcareous tufa.

The archaeological layers at Isernia La Pineta were discovered at *ca*. 4 m below the surface inside the older UBSU buried under sediments belonging to the Middle Pleistocene UNSU and represent the most complete sequence recognized in the area for these two units [22,30,31]. From the base to the top of the sedimentological sequence, five sedimentary units were recognized (Fig 2).

Units 5 and 4 both belong to the upper part of the oldest UBSU. Clayey lacustrine layers characterize Unit 5. Unit 4 is a phytoclastic and (to a minor extent) phytostromatic and phytohermal calcareous tufa deposited by a fresh water river. At the top of Unit 4, below the oldest occupation layer (t. 3c), a primary pyroclastic fall, here named U4T, including white weathered pumice (up to 1 cm in diameter) containing transparent sanidines, clinopyroxenes (Cpx) and biotites is found. The mineral composition is in accordance with products of shoshonitic and basaltic andesites affinities.

Units 3 and 2 of Isernia la Pineta belong to the second UBSU [23]. Unit 3 is composed of sands and thin layers of gravels deposited by ephemeral rivers and is subdivided into three sedimentary sub-Units (U3A, U3E, U3F see Fig 2). Sands are very rich in reworked volcanic material including sanidines and clinopyroxenes (Fig 2). An ^40^Ar/^39^Ar age of 610 ± 10 ka (2σ) was obtained on these reworked sanidines in the 3coll layer by Coltorti et al. [23]. Unit 2 is made of gravels with thin layers of sands deposited by ephemeral streams (Fig 2). The top of Unit 2 corresponds to a deeply weathered palaeosol (S2) (Fig 2). Finally, Unit 1 is a colluvium sequence, including a pyroclastic fall dated by ^40^Ar/^39^Ar at 499 ± 13 ka [23] (2σ) and weathered at the top by a palaeosol (S1) (Fig 2).

Four archaeosurfaces (3c, 3a, 3s10 sect. I, 3a sect. II) have been identified and excavated in the last 35 years. The oldest occupation layer (3c) lies at the top of calcareous tufa and is buried under a thin layer of mixed phytostromatic tufa and clay sediments. It is thus contemporaneous with Unit 4 [23] while in previous studies, it was erroneously assigned to Unit 3. The white pumice layer described above was sampled just inside the calcareous tufa below this archaeosurface (Fig 2).

In order to obtain a more precise chronology of the deposition sedimentary sequence, we dated single sanidine crystals using ^40^Ar/^39^Ar laser fusion method [32]. The ^40^Ar/^39^Ar method has been widely used in the last 25 years to provide an important radioisotopic control of human evolution because of its applicability to a period from an unlimited age to a few millennia. The single grain method provides assurance against the possibility that volcanic effluents could be contaminated with older minerals [33]. This method has recently allowed a better chronological approach to Italian fossil human remains [34].

The sanidines were extracted from both the tephra layer U4T and reworked Unit 3 volcanic material from layers 3coll, 3s10 and 3s6-9 located above the archaeosurface 3a (U3E, Fig 2). The youngest crystal population determined by ^40^Ar/^39^Ar will correspond to the age of the volcanic eruption and by extension to the age of the sedimentary layer when the dated material is a primary fall deposit (*eg*. U4T). When the deposit corresponds to a reworked volcanic sedimentary layer, the age of the youngest crystal population will correspond to the youngest eruption reworked (*eg*. units 3coll, 3s10 and 3s6-9) and in such area where numerous eruptive events are known, to the last eruption recorded before the sediment deposition.

## Methods

The systematic excavations in Isernia la Pineta are carried out by the University of Ferrara since the beginning with the authorization of the *Direzione Regionale per i Beni Culturali del Molise* and of the Italian Ministry of Culture. The archaeological material from Isernia la Pineta is conserved in the Museo Nazionale del Paleolitico (Isernia, Molise). The human remain has been labelled IS42 and is preserved for studies at the University of Ferrara, Department of Humanities (C.so Ercole I°d’Este, 32—Ferrara).

### Microtomography

Micro-CT data were obtained on the tooth with a Skyscan 1172 scanner using an X-ray tube voltage of 100 kV, a current intensity of 100 μA, an aluminium/copper filter and an exposure of 590 ms. The volume was reconstructed with an isometric voxel size of 9.11 μm. Segmentation of the micro-CT images was performed semi-automatically with manual corrections by Avizo 7.0.0 (Visualization Sciences Group Inc.)

### Morphological Description

The descriptive terminology and protocol follows Moggi-Cecchi et al. [35]. Non-metric traits were described using the Arizona State University Dental Anthropology System (ASU-DAS [35]) which, although devised for permanent teeth, allows for comparison with recent descriptions of other fossil hominin deciduous teeth (eg. [36,37]). Incisal wear was scored following Molnar [38]. Root resorption was recorded following Moorrees et al. [39]. Similarly, age at death was assessed combining different observations on tooth formation, root resorption and dental eruption [39,40].

### Metric Comparisons

Standard dimensions of the tooth were recorded with a digital calliper, i.e. the bucco-lingual diameter (BL); mesio-distal diameter (MD) corrected for interproximal attrition following Wood and Abbott [41] and the preserved crown height. These measurements were then compared with data from the literature dealing with Middle Pleistocene hominins (MPH), Neanderthals and modern humans [36,37,42–44]. Individual values from IS42 were referred to averages and standard deviations of the comparative samples with computed Z-scores.

Additional measurements were collected from the micro-CT images, such as the dimensions of small enamel hypoplasia pits, BL diameter of dentine exposure, dimension of the interproximal contact facets (IF) and dimensions of the root canal. The enamel thickness was measured on the buccal, lingual, mesial, and distal sides of the crown, at mid-crown. In detail, three virtual planes, parallel to one another, were defined: at the incisal edge, at the cervix on the lingual face and at halfway between the two. The third plane computed, corresponding to an hypothetical mid-crown plane (e.g. the wear stage being advanced, the crown is incomplete) was used for the estimation of the enamel thickness.

Unfortunately, comparative data available in the literature for MPH deciduous incisors are limited. Thus, the data obtained cannot be considered indicative of rather thin or thick enamel.

### ^40^Ar/^39^Ar dating

Pristine sanidine crystals ranging from 1 mm to up to 2 mm in size were extracted after crushing and sieving of the pumice extracted from the tephra layer from the upper part of U4. We also picked free sanidine crystals found in 3 coll, 3s10 and 3s6-9 units (fluvial units). All crystals were transparent and free of inclusions and were handpicked under a binocular microscope.

All selected crystals were slightly leached for 5 minutes in a 7% HF acid solution. After leaching, 30 crystals were handpicked for each sedimentary level as well as for the pumice layer and separately loaded in aluminium disks. The samples were irradiated for 30 minutes (IRR 66) for the pumice layer (U4T) and 60 minutes (IRR 83) for units 3coll, 3s10 and 3s6-9 in the β1 tube of the OSIRIS reactor (CEA Saclay, France). Argon isotopes were analysed using a VG5400 mass spectrometer equipped with a single ion counter (Balzers^®^ SEV 217 SEN) following procedures outlined in Nomade et al. [32]. Each Ar isotope measurement consisted of 20 cycles of peak switching of the argon isotopes. Neutron fluence (J) was monitored by co-irradiation of Alder Creek Sanidine (ACs-2 [45]) placed in the same pit as the sample. J values were determined from analyses of three ACs-2 single grains for each sample. We report ages relative to the ACs age of 1.193 Ma [45] and the decay constants of Steiger and Jäger [46]. Several proposed calibrations of the ^40^Ar/^39^Ar chronometer are currently in use, yielding ages that vary by ~2% in the time range of the M-B reversal [47–50]. However, this implied difference in calibrated age is about 12 ka and thus identical, within uncertainty, with the reported age.

The precision and accuracy of the mass discrimination correction was monitored by daily measurements of air argon at various pressures (see full experimental description [32]). Nucleogenic production ratios used to correct for reactor-produced Ar isotopes from K and Ca are given in S1 Table.

## Results and Discussion

### Morphology and Morphometry of IS42

**Fig 3:**
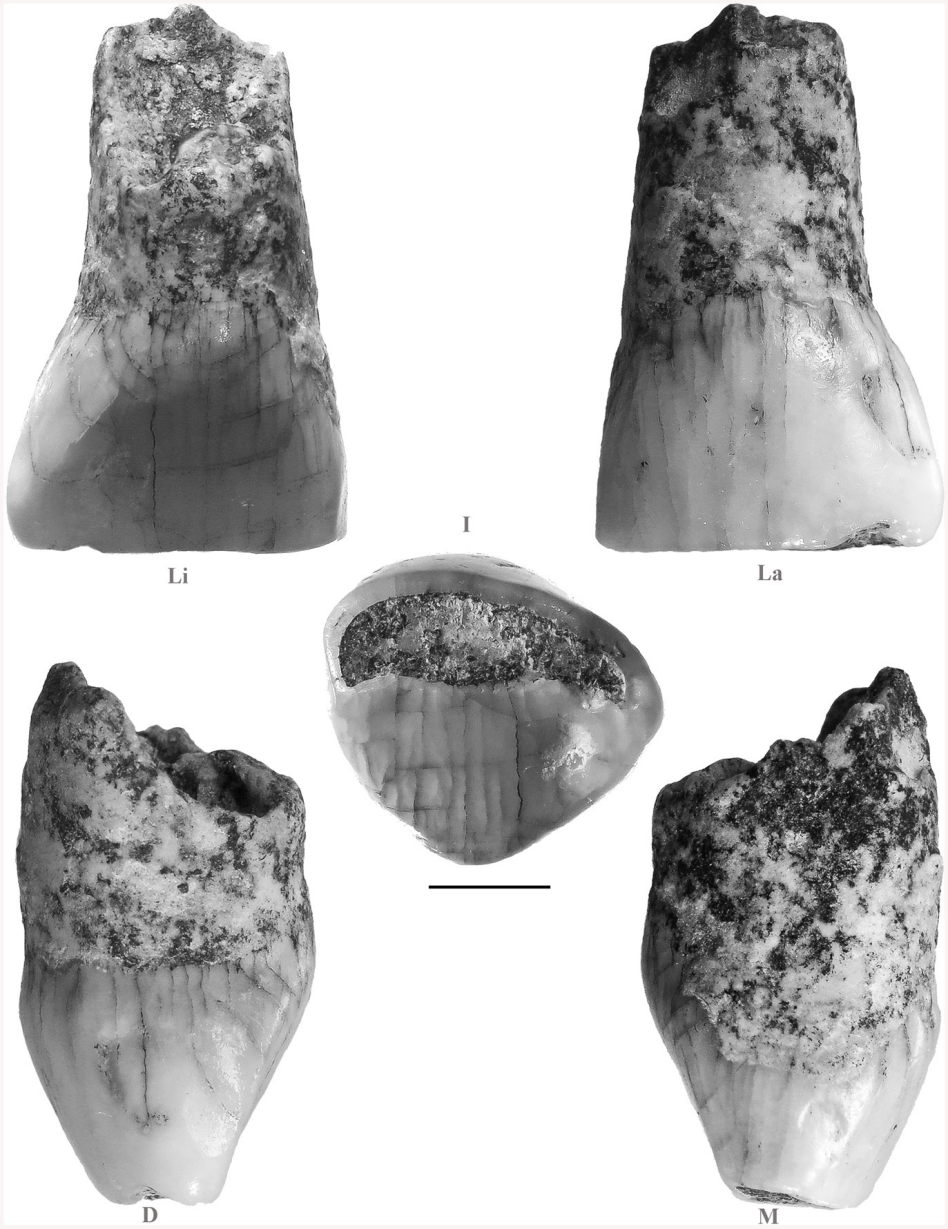
Isernia La Pineta deciduous incisor (IS42). Li: lingual; La: labial; I: incisal; D: distal; M: mesial. Scale bar 2 mm.

The tooth labelled IS42 is a well-preserved human specimen (Fig 3), including the crown and some 4 mm of the largely resorbed root. Incisal wear has exposed a large strip of dentine on the flat horizontal incisal edge (BL width 1.4 mm along the midline) corresponding to the 4th wear stage [38]. A tiny flake of enamel is missing from the incisal edge of the lingual face, near the distal corner. It is probable that this flake was detached post mortem, given the sharpness of its edges, while a larger flake of enamel that is missing from the incisal edge of the labial face shows a smoothed border, suggesting an ante-mortem loss due to functional wear. There is a small (inciso-cervical [IC] 1.1 mm / bucco-lingual [BL] 1.0 mm) and rounded mesial IF, which encroaches upon the incisal edge. The distal facet is also small and rounded (IC 1.2 mm / BL 0.9 mm), some 0.7 mm below the incisal edge and lingually placed. Localised encrustations are evident on the mesial face, extending both on the crown and the root in the area of the cervix.

The labial face has an almost straight IC profile, but with a marked MD convexity, distally skewed, corresponding to grade 3 of “labial convexity” in the ASU-DAS system. The outline is asymmetrical, with the mesial shoulder more angulated and the distal more rounded. The cervical enamel line is almost straight. Microscopically, the whole labial surface shows a large number of striations that we interpret as mainly due to functional wear.

On the lingual face the slight cervical eminence is symmetrical, corresponding to grade 1 of “*tuberculum dentale*” in the ASU-DAS system. Both the mesial and distal marginal crests are weakly developed, with the distal more marked. A weak mesial sulcus and distal sulcus can also be observed adjacent to the respective marginal crests. No “shovelling” is evident (grade 0 ASU-DAS).

Enamel defects in the form of pits of different size are observed on the labial and mesial face; the largest is located on the distal edge of the labial face (IC 1.5 mm and MD 1.0 mm).

The root is straight, oval in cross section, with a slight MD compression. The morphology of the exposed surface indicates a resorption process. The root is longer labially (4.8 mm) than lingually (2.5 mm) and the stage of resorption is slightly more than Res1/2 [39]. The root canal is small: 1.3 mm MD and 1.1mm LL.

Micro-CT images show that the enamel thickness at half of the preserved crown height is 0.29 mm on the labial face, 0.22 mm on the lingual face, 0.32 mm on the distal face and 0.15 mm on the mesial face. At the same level, the dentine is 3.55 mm LL and 5.32 mm MD.

Given its general features, IS42 is a deciduous incisor and, most probably (see also below), a deciduous maxillary left lateral incisor (di^2^), although it is not possible to exclude it might be regarded as either a maxillary left central incisor (di^1^) or a mandibular right lateral incisor (di_2_). Therefore, in comparing IS42 with other recent and fossil hominin specimens, we considered data on both di^1^, di^2^ and di_2_, as available in the literature.

Table 1 shows the non-metric traits examined (labial convexity, shovel shape, *tuberculum dentale*) and their degree of expression in IS42, compared with data on other specimens. However, to the best of our knowledge, comparative data in the literature using the ASU-DAS standard are only those for dental traits of di^1^ [37,38], whereas no consistent data on both di^2^ and di_2_ are available. These comparisons suggest marked differences with Neanderthals as far as the shovel shape and *tuberculum dentale* are considered; it is known, however, that Neanderthal deciduous incisors show a larger variability in terms of trait expressions than permanent incisors [51]. Nevertheless, IS42 is also different from Middle Pleistocene hominins (MPH), showing absence of shovel shape and a minimum degree of *tuberculum dentale*, while the degree of labial convexity is largely variable in MPH (as well as among Neanderthals and early modern humans).

**Table 1:** Non-metric morphological traits (ASU-DAS system [35]) of IS42 compared with other European specimens (di^1^).

	ASU UI/1 labial convexity	ASU UI/1 Shovel shape	ASU UI/1 *tuberculum dentale*
IS42	3	0	1
MPH^a^			
Terra Amata	-	-	3
Arago 23	3	5	3
Arago 58	2	1	0
Arago 80	3	5	3
Arago 92	3	5	3
Orgnac 7	3	3	2
Orgnac 8	1	-	-
Lazaret II	3	3	3
Lazaret V	1	1	0
Lazaret XI	0	1	0
N			
Fumane 4^b^	3	-	-
Le Portel 24^a^	2	3	2
Le Portel 28^a^	2	3	2
Pi8tel 28 2^a^	3	3	2
Manie 1^a^	2	3	2
Schoepflin 46^a^	3	3	2
EUP^a^			
Grotte du Renne 27	3	3	2
Grotte du Renne 32	2	-	2
Grotte du Renne 36	2	1	0

^a^ [36]

^b^ [37]

MPH = Middle Pleistocene hominins; N = Neanderthals; EUP = modern Europeans of the Upper Palaeolithic.

As for dental metric data, Table 2 reports the BL and MD diameters of IS42 compared with measurements taken on di^1^, di^2^ and di_2_ of other European fossil hominins [36,42–44]; descriptive statistics (including Z score values) are reported for some different groups: MPH, Neanderthals and modern humans, either of the Upper Palaeolithic or recent. Comparative data for MPH are available only for di^1^ dimensions. The Z-scores values of both BL and MD diameters of IS42 shows that the tooth from Isernia La Pineta falls within one standard deviation only for some dimensions of both di^1^ and di_2_, the closest affinities being with the Neanderthal di_2_ variability. Higher Z-score values for both BL and MD occur, among fossil hominins, when comparing IS 42 with Neanderthals di^2^, EUP di^1^ and Neanderthals di^1^.

**Table 2:** Dental dimensions of IS42 compared with other European hominin samples.

IS42			BL		MD			
			4.71		5.66			
		**di^1^**		**di^2^**			**di_2_**	
		**BL**	**MD**	**BL**		**MD**	**BL**	**MD**
MPH	*m*	5.34	7.34	-		-	-	-
	*s*	0.7	0.69	-		-	-	-
	*n*	8	10	-		-	-	-
	*Z score*	-0.9	-2.43	-		-	-	-
N	*m*	6.13	7.69	5.43		6.09	4.85	5.44
	*s*	0.35	0.36	0.39		0.27	0.25	0.52
	*n*	23	23	11		7	15	16
	*Z score*	-4.06	-5.64	-1.85		-1.59	-0.56	0.42
EUP	*m*	5.42	7.2	5.12		-	4.58	5.11
	*s*	0.35	0.52	0.23		-	0.3	0.32
	*n*	18	19	5		-	18	16
	*Z score*	-2.03	-2.96	-1.78		-	0.43	1.72
RMH	*m*	4.87	6.24	4.69		5.02	4.14	4.5
	*s*	0.35	0.46	0.35		0.29	0.37	0.47
	*n*	47	47	37		30	62	63
	*Z score*	-0.46	-1.26	0.06		2.21	1.54	2.47

MPH = Middle Pleistocene hominins; N = Neanderthals; EUP = European Upper Palaeolithic modern humans; RMH = recent modern humans (*m*: mean, *s*: standard deviation, *n*: sample size; IS42 *Z scores* with respect to dimensions of each OTU are also reported) [36,42–44].

### New Chronological Constraints

^40^Ar/^39^Ar single crystal laser fusion was applied to sanidines from four different levels including the one extracted from juvenile pumice found in U4T directly below the archaeosurface 3c and the reworked ones extracted from pumice clasts from three sedimentary layers belonging to U3E (3coll, 3s10 and 3s6-9). Full analytical details for individual crystals from each level are given in the Supporting Information. In fig 2 we present the results as probability diagrams [33]. The youngest homogeneous population of sanidine crystals was calculated when the weighted mean age of these crystals has the following statistical characteristics: Mean Square Weighted Deviation (MSWD) < 1.5, Probability of fit> 0.1.

**Fig 4:**
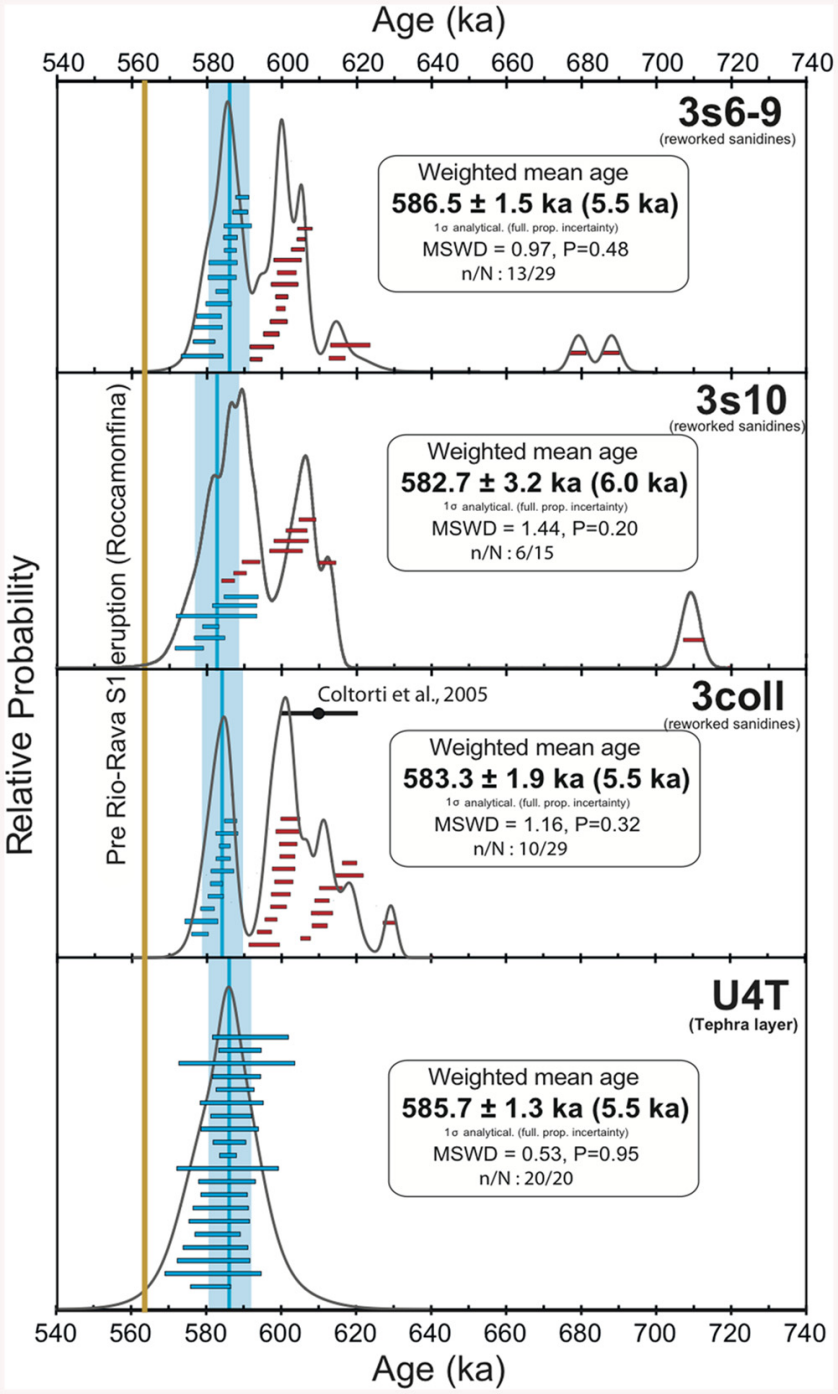
Probability diagrams for the four dated units at the Isernia La Pineta archaeological site. Error bars for individual measurements are reported at 1σ confidence level. Weighted mean ages are relative to ACS-2 at 1.194 Ma and given at 1σ analytical (fully propagated uncertainty). For comparison we report the age given in Coltorti et al.[23] for unit 3 coll. The pre-Rio Rava S1 eruption age (brown line) is estimated according to Giaccio et al.[57] and constitutes the terminus *post quem* age of the Isernia La Pineta site.

The probability diagram obtained for the crystals from the U4T tephra layer is simple, dominated by primary crystals (Fig 4) allowing us to calculate a precise weighted mean age of 586 ± 1 ka (1σ analytical uncertainty, J = 0.00035760 +/- 0.00000122). The absence of xenocrystal contamination reinforces the primary character of this layer. This age is interpreted as the age of deposition of the pumice as well as Unit 4.

As expected, probability diagrams obtained for sanidine crystals coming from the fluvial levels (U3E, layers 3coll, 3s10 and 3s6-9) show a wide range of ages between 709 and 576 ka and multimodal probability diagrams (Fig 4). The ages of 583 ± 2 ka (J = 0.00042480 +/- 0.00000093), 583 ± 3 ka (J = 0.00041880 +/- 0.00000188) and 587 ± 2 ka (J = 0.00041630 +/- 0.00000129) obtained for the youngest sanidine populations for layers 3 coll, 3s10 and 3s6-9 respectively (Fig 4) are identical within uncertainty with the age of the U4T tephra. These results suggest that these sanidines represent reworked material from the primary underlying tephra layer U4T in Unit 4. These youngest homogeneous populations of crystals are interpreted as the last source of volcanic material reworked in each fluvial layer. The very high analytical precision provided for each dated sanidine crystal in the fluvial layers we investigated demonstrates that the age of 610 ± 10 ka suggested by Coltorti et al. [23] was slightly overestimated because of unrecognized older crystals included in the weighted mean age calculation. These measurements were performed on single grains and on populations of two to five crystals with a VG 3600 spectrometer equipped with an Faraday cup detector. The facts that previous data were not done solely on single crystal and that the detector used was less sensitive than our ion counter probably explain why the population at 610 ka seemed homogeneous at the time.

## Conclusions

### IS42 Attribution and Taxonomy

We identified the new fossil tooth from Isernia La Pineta as a human deciduous incisor because of the single root, partly resorbed, and a MD diameter elongated in the incisal margin of the crown. In addition, the marked MD convexity of the labial face and the asymmetric profile of the crown, distally skewed in incisal view suggest that the tooth is a deciduous left maxillary incisor.

It is more difficult to ascertain whether the specimen is a central or lateral maxillary incisor. Features that point towards an attribution as di^1^ include MD diameter larger than crown height (reconstructed), position of the mesial and distal interproximal contact facets and relatively marked development of the *tuberculum dentale*. At the same time, the asymmetrical outline in labial view, the marked distal skewing and the small dimensions suggest an attribution as di^2^. By contrast, the comparative analysis of dental dimensions, with reference samples attributed to di^1^, di^2^ and di_2_, suggests affinities also with a deciduous mandibular lateral incisor. However, the lack of a consistent comparative sample belonging to MPH hominin, particularly for both di^2^ and di_2_, prevents any clear identification based on morphometric data only.

We believe that the combined evidence points towards an attribution to the di^2^ tooth class, although both the other alternatives (di^1^ and di_2_) cannot be completely ruled out, given that the comparative data set is largely composed of human groups other than MPH, that is Neanderthals and modern humans. We suggest a di^2^ attribution mostly based on morphological observations, which include, as mentioned above, the asymmetrical outline, the marked distal skewing, the convexity of the labial face (usually lacking in deciduous mandibular lateral incisors), the lack of a flattening of the crown in the BL direction (usually present in deciduous mandibular lateral incisors).

Given this interpretation, the degree of root resorption (Res ½) point to an age of 5–7 years according to modern human standards [39,40]. The relatively extended portion of root still preserved might suggest that the tooth was still in the mouth; in this event, the reconstructed age would correspond to the age at death of the individual.

A firm taxonomic identification of IS42 is not possible at present, given the paucity of comparative samples and the variability observed among MPH as well as within the Neanderthals. Given the emerging variegated pattern of hominin evolution during the Middle Pleistocene in Europe [52–55], the most conservative option is attributing the tooth to an undetermined species of the genus *Homo*, i.e. to *Homo* sp. (cf. *heidelbergensis*).

### About the Age of the Three Layers Bearing the Archaeological Remains

From a climatostratigraphic point of view, the calcareous tufa found in Unit 4 is associated with fresh water bodies and mostly forested slopes usually deposited during interglacials or, more rarely, during interstadials, while the subsequent sands and gravels (*ca* Unit 2) reveal the onset of cold conditions with bare slopes affected by gelifraction processes [23].

**Fig 5:**
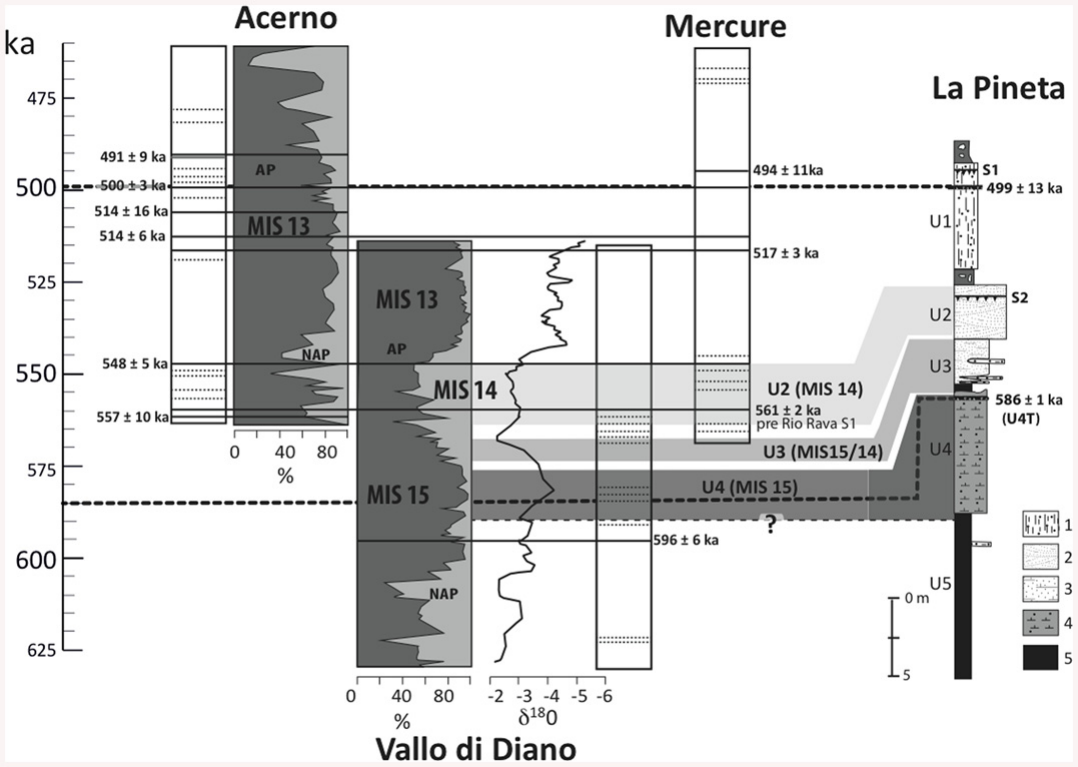
La Pineta stratigraphy and ages are compared to the well-known tephrostratigraphy and palaeoenvironmental records of three Southern Apennines basins (Acerno, Vallo di Diano, Mercure). Black lines are for ash layers dated by ^40^Ar/^39^Ar, dash lines for recognized ash layers but not dated. Bold dash lines are the ages obtained for the primary volcanic deposit (U4T) at Isernia la Pineta site from this study and in Unit 2 [23] Southern Apennines data are from Karner et al., 1999; Giaccio et al., 2014; Petrosino et al., 2014 [56,57,62]. All ^40^Ar/39Ar ages are recalculated according to the same standard ACS-2 at 1.193 Ma [45].

The age of 586 ± 1 ka obtained for U4T can be considered as a maximum age of the archaeological layers found in Unit 3. As the sedimentological observations suggest that Unit 4 was deposited during a warm stage (interglacial or interstadial) and thanks to the new ^40^Ar/^39^Ar ages, it can be concluded that Unit 4 was deposited during interglacial MIS15 (Fig 5). The age of U4T fits well with the cluster of prevailing phonolithic eruptions recognized in the Vallo di Diano lacustrine succession [56]. This group of eruptions spans the various MIS 15 substages [56] (Fig 5). During this time period both Roccamonfina and Monte Vulture volcanoes were high-explosively active. Due to the large grain sizes of pumice (up to 2 mm) and phenocrystals a relatively proximal source of tephra U4T is proposed. Roccamonfina stratovolcano is the closest volcanic source and in a favourable wind position to the Isernia basin (35 km South-West of Isernia la Pineta) compared to the Monte Vulture volcano, which is located 130 km towards the North-West. A further evidence for Roccamonfina as a source volcano is the mineralogical assemblage of U4T that indicates a phonolitic affinity (sanidine, clinopyroxene, biotite) with a lack in leucite crystals, which are common in the Monte Vulture tephras. [57]. Unfortunately, the U4T tephra is slightly weathered and the volcanic glass is transformed into clays, which made the chemical analysis for further detailed characterisation and identification of this tephra layer currently impossible. The oldest exposed products of Roccamonfina stratovolcano have an age of about 560 ka [57]. However, Peccerillo [58] stated that “*tephra recovered from the nearby areas have shown a somewhat older age of about 580 ka*, *which most probably represents the beginning of the volcanic activity at Roccamonfina*".

The age of Unit 3 is more complex to estimate; however we suggest that the age of the level 3coll, where the human remain was found, and of the four archaeological levels to be at the transition between interglacial MIS 15 and glacial MIS 14, more precisely between 583 ka (Unit 4 deposition age) and *ca*. 561 ka, at the beginning of the full glacial MIS 14 (Fig 5) for the following reasons: 1) Unit 3 was deposited at the onset of cold conditions as demonstrated by Coltorti et al.[23]; 2) small and large mammals assemblage, in particular the presence of *Arvicola mosbachensis* with archaic characters [3,59]; 3) this same assemblage point towards more arid and cooler conditions than at present with an arboreal steppe [2,3]; 4) ^40^Ar/^39^Ar ages obtained in the Unit 1’s pyroclastic fall (499 ± 13 ka; [23]) 5 m above the archaeological levels indicate that the upper part of the basin was deposited during the next interglacial MIS 13 (Fig 5); 5) the absence of sanidine grains younger than 576 ka in the analysed populations (n = 73) extracted from three layers bearing the archaeological remains within Unit 3 suggests that the deposition of these layers predates several known eruptions of the early activity of the Roccamonfina such as the pre-Rio Rava eruption (~561 ka; [57]). The pre-Rio Rava eruption is an important regional marker as this eruption is synchronous with the onset of the full cold stage 14 in several Apennines basins (*ca*. Acerno, Mercure; see Giaccio et al. [57] and Fig 5).

Therefore the human occupations found within Unit 3 occurred at the transition between an interglacial/interstadial to Glacial/Stadial (Fig 5) (*ca*. MIS15/MIS14).

It is worth mentioning the fact that the level 3coll is a fluviatile reworked deposit and it might be possible that the human tooth comes from the archaeological layer t 3a just below. Between both the levels UT4 and 3coll lies a large sterile silty-clay layer (1 m thick) and thus the tooth cannot be older than this layer. This observation have no impact on the age of the tooth we proposed above.

### Final Remarks

**Fig 6:**
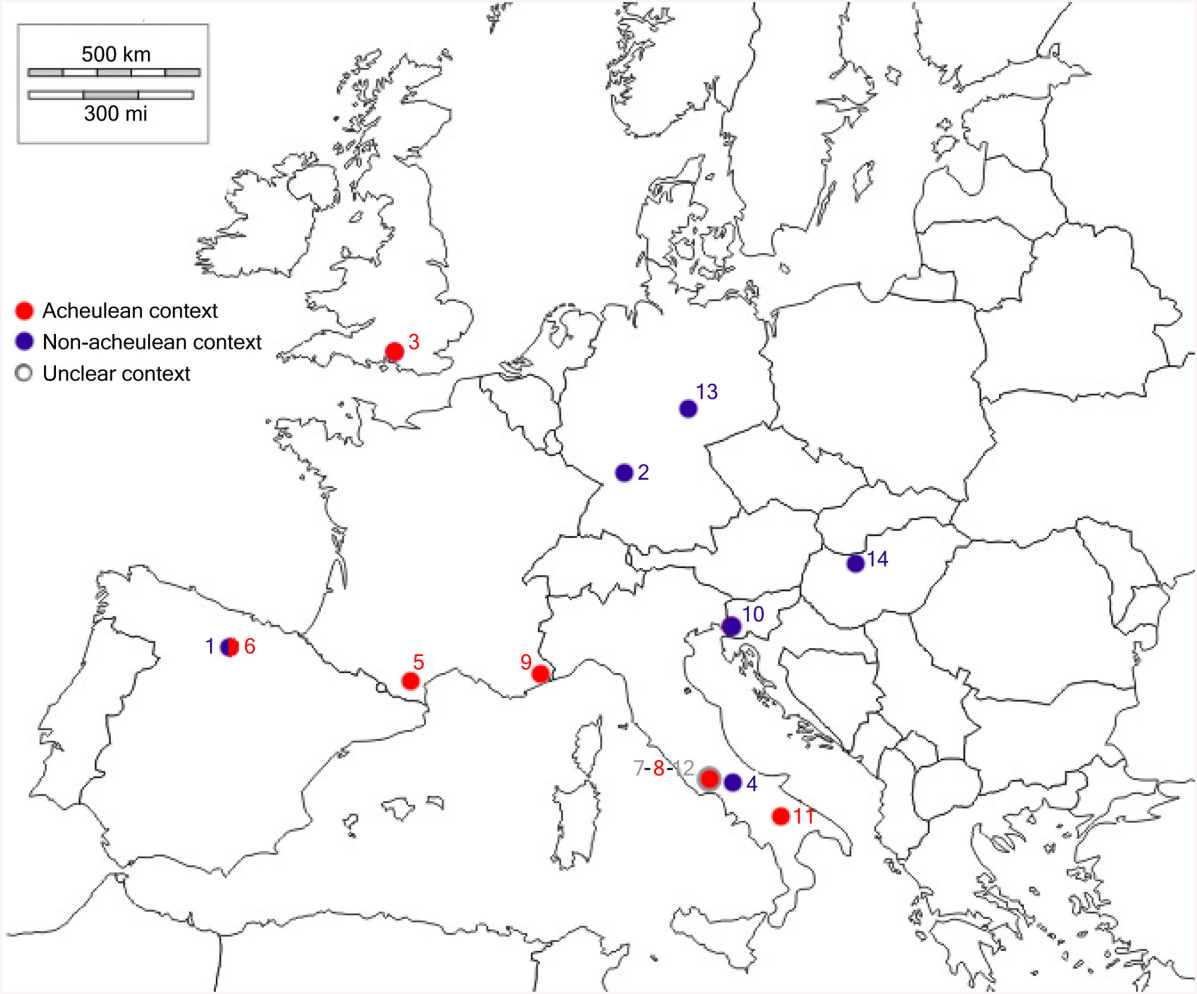
European sites with human remains dated to ca. 800 to 350 ka VS. the presence or absence of Acheulean context. 1. Gran Dolina TD6 [63–65]; 2. Mauer [18,66–68]; 3. Boxgrove [13,69–71]; 4. Isernia La Pineta; 5. Caune de l’Arago [10,72]; 6. Sima de los Huesos [73–76]; 7. Ceprano [60,61]; 8. Fontana Ranuccio[77,78]; 9. Terra Amata [36,79,80]; 10. Visogliano [81–83]; 11. Venosa–Notarchirico [9,84]; 12. Pofi [85,86]; 13. Bilzingsleben [87,88]; 14. Vértesszõlõs [89,90].

Combining the new chronological data with the archaeological evidence collected at Isernia La Pineta, we note that this site of the end of MIS 15 is characterized by the absence of handaxes, pivotal elements that occur in most of the other European archaeological sites of the Middle Pleistocene (Fig 6).

Looking at the human fossil record of the same time slab, a large variability is observed in the morphology of the human fossil specimens, with persistence of archaic features such as those displayed by the calvarium from the nearby site of Ceprano [60,61]. These observations and the archaeological evidence of Isernia La Pineta may suggest the occurrence of ecological “refugia” in south-central Italy until at least MIS 11.

The new human fossil tooth from Isernia La Pineta represents a small but significant addition to the very limited human fossil record in the Middle Pleistocene. It also provides further evidence of the morphological variability displayed by MPH. It may be thus regarded in the framework of such a complex scenario (e.g., [53]) as a deciduous incisors belonged to a child of about 5–7 years that shared only some features with the polymorphic variability of *Homo* cf. *heidelbergensis* known so far.

## Supporting Information

S1 File 40Ar/39Ar detailed results. (DOCX)

S2 File Petrographic Analysis. (DOCX)

S1 Table 40Ar/39Ar detailed results. Location corresponds to the position of the sample in the excavation area: l = quadrants number inside the pavilion (first excavation area); S: layer number; q: square number.
(XLS)
