# Supplementary material for: A Human Deciduous Tooth and New 40Ar/39Ar Dating Results from the Middle Pleistocene Archaeological Site of Isernia La Pineta, Southern Italy
Source: PLoS One. 2015 Oct 12;10(10):e0140091. doi: 10.1371/journal.pone.0140091 (PMC4601758; doi:10.1371/journal.pone.0140091)
Supplement: S1 File — (DOCX) [file pone.0140091.s001.docx]

**^40^Ar/^39^Ar detailed results (Tab. 1-4, supplementary)**

Data reduction and isochron regressions were calculated using ArArCalc (Koppers, 2002). Weighted mean age and corresponding uncertainties were calculated using IsoPlot 3.0 (Ludwig, 2001) and are given at the 1s level (Renne et al., 2009). Fully propagated uncertainties are also provided for each sample. Results for each unit are discussed below from the lowermost stratigraphic unit to the highest.

***U4 unit***: Twenty crystals were analyzed. The age-probability density spectrum is simple, with one prominent mode, suggesting that all analyzed crystals belong to the same population (Fig. 2). A weighted mean age of 585.7 ± 1.3 ka (MSWD = 0.53, P= 0.95, 1s analytical uncertainty, 5.5 ka full propagated uncertainty) was obtained for this homogeneous population. The inverse isochron plot age based on all crystals is identical (i.e. 584.7 ± 2.9 ka (MSWD =0.54)). The initial ^40^Ar/^36^Ar intercept (304.5 ± 15.4) is, within uncertainty, identical to the atmospheric ratio, suggesting no excess argon component.

***3 coll unit***: Twenty-nine crystals were analyzed in two distinct parcels: fourteen crystals from parcel I1 P106 and fifteen crystals from parcel I4 P101. A single age-probability density spectrum was built by combining the two parcels (Fig. 3). This spectrum displays multiple modes, suggesting that the sanidines from this unit belong to various populations. The youngest mode represented by ten crystals gives a weighted mean age of 583.3 ± 1.9 ka (MSWD = 1.8, P= 0.06, 1s analytical uncertainty, 5.5 ka 1s full propagated uncertainty). The corresponding isochron plot age based on all crystals from the youngest mode is identical (i.e. 582.6 ± 3.7 ka (MSWD =1.8)). The initial ^40^Ar/^36^Ar intercept (295.8 ± 2.2) is, within uncertainty, identical to the atmospheric ratio, suggesting no excess argon component. The age we obtained is significantly younger than the age reported by Coltorti(Coltorti et al., 2005) for the same unit (i.e. 610 ± 5 ka). However, the age discrepancy is due to the fact that the individual uncertainty for each crystal was much larger than for our new measurements, which did not allow the analysis to reveal the various populations of crystals.

***3s10 unit***: Fifteen crystals were analyzed from parcel I4 P106 (Fig. 3). The age-probability density spectrum displays several modes; the youngest one, represented by six crystals, gives a weighted mean age of 582.7 ± 2.3 ka (MSWD = 1.4, P= 0.19, 1s analytical uncertainty, 6.0 ka 1s full propagated uncertainty). The inverse isochron plot age based on all crystals of 582.6 ± 3.7 ka (MSWD =1.8) and the ^40^Ar/^36^Ar intercept (295.8 ± 2.2) identical to the atmospheric ratio suggest no excess argon component.

***3s6-9***: Twenty-nine crystals were analyzed in two distinct parcels: fourteen crystals from parcel I1 P106 and fifteen crystals from parcel I4 P101. A single age-probability density spectrum was built combining the two parcels (Fig. 2). This spectrum displays multiple modes, showing that the sanidines come from several populations. The youngest mode, represented by thirteen crystals, gives a weighted mean age of 586.0 ± 1.6 ka (MSWD = 1.4, P= 0.18, 1s analytical uncertainty, 5.5 ka 1s full propagated uncertainty). The corresponding isochron age based on all crystals from the youngest mode (582.6 ± 3.7 ka (MSWD =1.8)) and the initial ^40^Ar/^36^Ar intercept identical to the atmospheric ratio (i.e. 295.8 ± 2.2) suggest no excess argon component.

**References**

Coltorti M, Féraud G, Marzoli A, Ton-That T, Voinchet P, Bahain J-J, Minelli A, Thun Hohenstein U, Peretto C. 2005. New 40Ar/39Ar, stratigraphic and palaeoclimatic data on the Isernia la Pineta Lower Palaeolithic site, Molise, Italy,. Quat Int 113:11–22.

Koppers AAP. 2002. ArAr CALC—software for 40Ar/39Ar age calculations. Comput Geosci 28:605–619.

Ludwig K. 2001. Isoplot 3.0—A geochronological toolkit for Microsoft Excel. Berkeley G. Berkeley.

Renne PR, Deino AL, Hames WE, Heizler MT, Hemming SR, Hodges K V., Koppers AAP, Mark DF, Morgan LE, Phillips D. 2009. Data reporting norms for 40Ar/39Ar geochronology. Quat Geochronol 4:346–352.
