# Supplementary material for: A Human Deciduous Tooth and New 40Ar/39Ar Dating Results from the Middle Pleistocene Archaeological Site of Isernia La Pineta, Southern Italy
Source: PLoS One. 2015 Oct 12;10(10):e0140091. doi: 10.1371/journal.pone.0140091 (PMC4601758; doi:10.1371/journal.pone.0140091)
Supplement: S2 File — (DOCX) [file pone.0140091.s002.docx]

The most part of sediment in Isernia la Pineta is characterized by cryptocrystalline calcite originated by chemical precipitation (up to 55% of the mineral phases forming the rock) that host pyroclastic components with highly variable wealth. The Loss on Ignition (L.O.I.) is strongly related with the CaO so the low H2O amount (< 5%), also in the colluvium strata, is related to unaltered very fine volcanic ash.

**Figure 1: CaO percentage and Loss on Ignition (L.O.I) correlation.**

The silicate matrix of the sediments has a clear volcanic origin with petrologic potassic affinities containing a trace elements pattern similar to those of shoshonite volcanoes in the Campanian area and in Volcanic Roman Province. The pattern isn’t modified by sedimentary contributions and changes in the geochemical fingerprint are absent. Therefore, the deposit can be compared with the products of volcanic eruption in the neighbouring areas from the same chronological range. Only the colluvium strata have a small anomalous amount of Th, Ce, P and Y that can be related to little pedogenesis contribution (sample P10 of fig. 2)

**Figure 2: Trace element values normalised with CI chondrite (McDonough and Sun, 1995)**. The provenience of the sample from top to base: P12, P13 and P11D: U3A - SU3S1-5; P11C and P11B: U3E - SU3S6-9; P10: U3E - SU3coll; P14: U3F - SU3a base; P4 and P2: U3F - SU3b; P1: U4 - SU3C (see Fig.2 from main manuscript).

The trace element values normalised with CI chondrite (McDonough and Sun, 1995, Fig. 2) have high LIL/HFS element values and negative anomalies of K, Nb, P, Ti; typical of High Potassium Calcalcaline and of Shoshonite Volcanic products of Roccamonfina (Conticelli et al., 2008, 2013). The tectonic setting of the ultrapotassic rocks in Isernia La Pineta has a geochemical signature typical of volcanic active continental margin (CAP, Muller and Groves, 1997).

The petrographic analysis confirm the presence of magmatic components in the silicate fraction and in the studied levels we found: 1) fragments of volcanic rock (sientiti), polycrystalline aggregates (glomeruli) and idiomorphic phenocrysts of potassium feldspar, plagioclase, pyroxene and phlogopite augutico-salitici titanite and often immersed in a glass matrix. 2) highly vesicular pumice, 3) fragments of metamorphic rocks (marbles in phlogopite). The colluvium has very fine dimensions of volcanic ashes only observable with SEM.

The chemical composition of the silicate component corresponding to the volcanic products of Roccamonfina although if they are less rich in magnesium, Cr, Ni, Co, V, probably because of the gravitational selection effect induced by the significant distance from the vents or by the local provenience. These volcano contributions to the sedimentary sequence of Isernia la Pineta are similar to the first phase of composite volcano Roccamonfina whose activity begun at 630ka (Ballini et al., 1989a; b; Conticelli et al., 2008), even if some geochemical differences are not explicable by a simple gravitational selection or pedogenesis processes. Consequently, we cannot exclude a contributions from local eruptions of fissure events.

**References**

Ballini A, Barberi F, Laurenzi M, Mezzetti F, Villa I. 1989a. Nuovi dati sulla stratigrafia del vulcano di Roccamonfina. Boll Grup Naz Vulcanol 5:533–555.

Ballini A, Frullani A, Mezzetti F. 1989b. La formazione piroclastica del tufo trachitico bianco del vulcano di roccamonfina. Bollettino Gruppo Nazionale Vulcanologia. Boll Grup Naz Vulcanol 5:557–574.

Conticelli S, Marchionni S, Rosa D, Giordano G, Boari E, Avanzinelli R. 2008. Shoshonite and sub-alkaline magmas from an ultrapotassic volcano: Sr–Nd–Pb isotope data on the Roccamonfina volcanic rocks, Roman Magmatic Province, Southern Italy. Contrib to Mineral Petrol 157:41–63.

McDonough WF, Sun S -s. 1995. The composition of the Earth. Chem Geol 120:223–253.

Muller D, Groves DI. 1997. Potassic Igneous Rocks and Associated Gold-Copper Mineralization. Berlin/Heidelberg: Springer-Verlag.
